# Neurofunctional and neuroimaging readouts for designing a preclinical stem-cell therapy trial in experimental stroke

**DOI:** 10.1038/s41598-022-08713-z

**Published:** 2022-03-18

**Authors:** Chloé Dumot, Chrystelle Po, Lucille Capin, Violaine Hubert, Elodie Ong, Matthieu Chourrout, Radu Bolbos, Camille Amaz, Céline Auxenfans, Emmanuelle Canet-Soulas, Claire Rome, Fabien Chauveau, Marlène Wiart

**Affiliations:** 1 Univ Lyon, CarMeN Laboratory, Inserm U1060, INRA U1397, INSA Lyon, Université Claude Bernard Lyon 1, Université Claude Bernard Lyon 1, Lyon, France; 2 Hospices Civils de Lyon, Lyon, France; 3 ICube, Université de Strasbourg, CNRS, UMR 7357, Strasbourg, France; 4 Tissue and Cell Bank, HCL, Lyon, France; 5 Univ Lyon 1, Lyon Neurosciences Research Center, CNRS UMR5292, Inserm U1028, Université Claude Bernard Lyon 1, Université Claude Bernard Lyon 1, Lyon, France; 6 Cermep, Lyon, France; 7 Clinical Investigation Center, CIC 1407, HCL, Louis Pradel Hospital, Lyon, France; 8 Inserm, U1216, Grenoble Institut des Neurosciences (GIN), Université Grenoble Alpes, 38000 , Grenoble, France; 9 CNRS, Lyon, France; 10 U1060 CARMEN-IRIS Team, Groupement Hospitalier Est, Bâtiment B13, IHU OPERA, 59 Boulevard Pinel, 69500 Bron, France

**Keywords:** Preclinical research, Stroke, Stem-cell research, Translational research

## Abstract

With the aim of designing a preclinical study evaluating an intracerebral cell-based therapy for stroke, an observational study was performed in the rat suture model of ischemic stroke. Objectives were threefold: (i) to characterize neurofunctional and imaging readouts in the first weeks following transient ischemic stroke, according to lesion subtype (hypothalamic, striatal, corticostriatal); (ii) to confirm that intracerebral administration does not negatively impact these readouts; and (iii) to calculate sample sizes for a future therapeutic trial using these readouts as endpoints. Our results suggested that the most relevant endpoints were side bias (staircase test) and axial diffusivity (AD) (diffusion tensor imaging). Hypothalamic-only lesions did not affect those parameters, which were close to normal. Side bias in striatal lesions reached near-normal levels within 2 weeks, while rats with corticostriatal lesions remained impaired until week 14. AD values were decreased at 4 days and increased at 5 weeks post-surgery, with a subtype gradient: hypothalamic < striatal < corticostriatal. Intracerebral administration did not impact these readouts. After sample size calculation (18–147 rats per group according to the endpoint considered), we conclude that a therapeutic trial based on both readouts would be feasible only in the framework of a multicenter trial.

## Introduction

Ischemic stroke is a leading cause of mortality and disability worldwide^1^. To date, the only therapeutic option is to reopen the occluded artery mechanically and/or pharmacologically^2^. This option is applicable only in the acute phase for selected patients. In case of persisting disability, there is no treatment in the chronic phase to restore function, except rehabilitation.

Stem-cell therapy is a promising therapeutic option to restore function in the acute, subacute and chronic phases of ischemic stroke^3–5^. Mesenchymal stem cells are of major interest due to their low immunogenicity profile, good availability and absence of ethical concerns. These pluripotent cells have the capacity to differentiate into different cell types but their use is mainly based on their immunoregulatory properties. Adipose mesenchymal stem cells (ASCs) are the more accessible source compared to bone marrow mesenchymal stem cell^6,7^. Human adipose-derived mesenchymal stem cells (hASCs) are already used in stroke clinical trials (NCT03570450)^8^. However, the optimal route, time-window and cell dose still need to be determined in well-designed preclinical studies. Intracerebral injection seems to be the most efficient route in terms of preclinical treatment efficacy due to the direct delivery of stem cells but was mostly studied using cells of neural origins^9^. Although mesenchymal stem cells have been shown to differentiate into neurons^10^, the main rationale for administering them intracerebrally is to take advantage of their effects locally on the microenvironment, with the hope that it may foster regeneration, for instance by promoting neuronal stem cell migration and differentiation, producing trophic factors and modulating neuroinflammation^11^. Recent phase 0/1 clinical trials have also reported the safety of this administration route in patients in the chronic stage of stroke^8,11,12^. In this context, our global aim was to design a preclinical study to evaluate the effects of intracerebral administration of clinical-grade hASCs in ischemic stroke, with a study design that aligns with clinical functional evaluation methods for long-term recovery studies^3,13^.

As is well-known, there are several obstacles to the translation of stem-cell research in ischemic stroke from the preclinical to the clinical arena. The rigor of study design, the inclusion of different stroke subtypes, the choice of appropriate primary readout parameters and well-defined sample sizes have been identified as key factors to overcome the translational roadblock^13–17^. The assessment of neurofunctional outcome in chronic stroke patients relies on clinical scores such as the National Institutes of Health Stroke Scale (NIHSS)^8^ and the upper limb movement section of the Fugl–Meyer (FM) scale^18,19^. Combining clinical scores with the assessment of ipsilesional corticospinal tract (CST) remodeling with diffusion tensor imaging (DTI) can improve prediction of motor outcome^19–22^. Accordingly, our preclinical stem-cell trial aimed at combining neuroscores and the staircase test, a skilled reaching task that assesses forelimb function in rodent models^14^, with the DTI evaluation of CST structural integrity (internal capsule).

There is a plethora of studies in the literature that evaluate stem cell therapy in rodent models of ischemic stroke using neurofunctional and imaging outcomes^5^. However, despite international recommendations^14,23^, most of them do not perform a priori sample size calculation and include a limited number of animals per group (sometimes down to 5–6 animals per group^24,25^). Such studies are very likely to be underpowered^26^. Because of the bias to publish positive results only, this might result in overstatement of efficacy^27^. In this context, there is a need to improve the methodology of therapeutic preclinical trials. Ideally, a rigorous study design implies to thoroughly investigate these endpoints according to stroke subtype, in order to determine the optimal frequency of measurements, the post-stroke period during which data should be monitored, the quantitative modifications of readouts in time, and the within-laboratory variances. The specific aims of the present observational study with a limited number of subjects were threefold: (i) to characterize neurofunctional readouts (neuroscores and staircase test) and DTI metrics in the first weeks following transient middle cerebral artery occlusion (tMCAO) according to stroke subtype; (ii) to confirm that intracerebral administration of hASCs does not negatively impact these readouts (because of the invasiveness of the procedure); and (iii) to determine the most appropriate functional and imaging endpoints, at which time-points they should be evaluated, and to calculate the sample size required to achieve statistically significant differences with these endpoints for a preclinical exploratory therapeutic trial.

## Results

**Figure 1:**
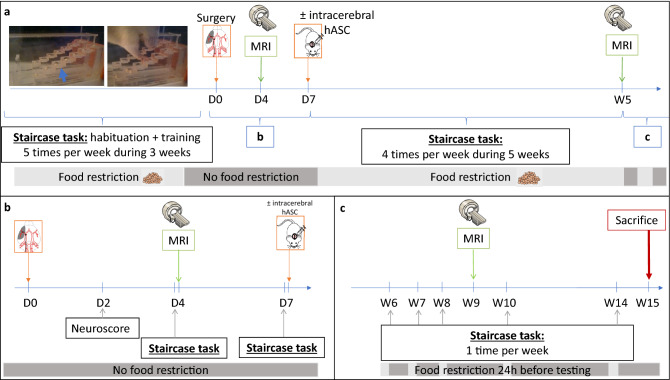
Study design. (**a**) Experimental design; (**b**) focus on the first week of the experiment; (**c**) focus on weeks 6 to 15: extended follow-up only for rats with corticostriatal lesions. *D* days, *MRI* magnetic resonance imaging, *hASC* human adipose mesenchymal stem cells, *W* Weeks.

Figure 1 shows the experimental design of the study. Briefly, after a 3-week period of training at the staircase task, transient (60 min) middle cerebral artery occlusion was performed at day 0 (D0) (Fig. 1a). Neuroscores were obtained at D2 post-surgery (Fig. 1b). The staircase test was then performed at D4 (before baseline MRI) and D7 (before treatment administration). Baseline MRI, including T2-weighted imaging (T2WI) and diffusion tensor imaging (DTI) sequences, was performed at D4 post-surgery (Fig. 1b). Cerebral lesions were stratified into 3 subtypes according to their location on baseline MRI: corticostriatal, striatal or hypothalamic-only^28^. Half of the rats received clinical-grade hASCs intracerebrally at D7. All rats were then monitored for 5 weeks with longitudinal neurofunctional tests and MRI. By this time, most rats had completely recovered according to neurofunctional testing, except those with corticostriatal lesions; for these rats, follow-up was extended to week 14 (W14) (Fig. 1c).

### Stroke subtypes

**Figure 2:**
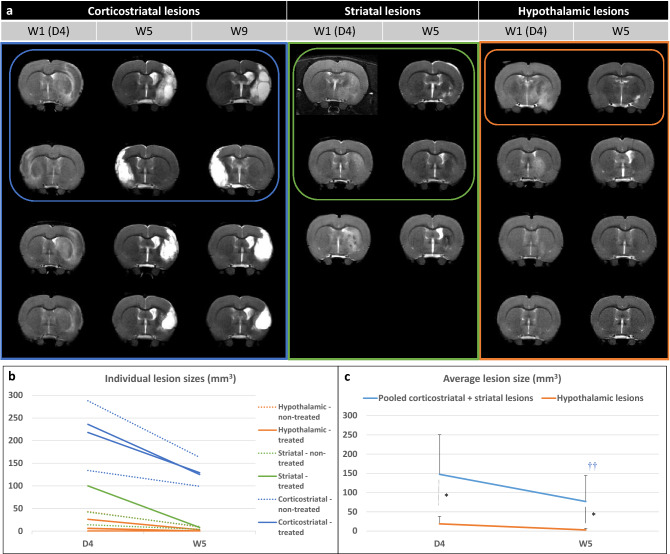
Evaluation of lesions on T2-weighted imaging. (**a**) Longitudinal T2-weighted imaging of all included rats according to lesion subtype (only one central slice is shown). Treated rats (that received intracerebral administration of hASCs) are presented in top rows and circled. (**b**) Individual lesion sizes are presented according to lesion subtype (striatal, corticostriatal and hypothalamic lesions) and treatment group (plain line: treated; dashed line: non-treated) at day 4 (D4) and week 5 (W5) post-surgery. (**c**) Average lesion sizes are presented according to lesion subtype: pooled (corticostriatal + striatal) vs hypothalamic lesions. Data are displayed as mean ± SD. *W* weeks, *D* days; *p < 0.05, (corticostriatal + striatal) vs hypothalamic, Wilcoxon–Mann–Whitney test; ^†^p < 0.05, ^††^p < 0.01, W5 vs D4, Friedman test.

Supplementary Figure S1 presents the CONSORT-like chart of the study. Of the 25 rats trained at the staircase test, 18 matched the inclusion criteria and were thus selected to undergo surgery. Seven rats died before the end of the experiment: 2 during the surgical procedure, 4 in the first 24 h and 1 in the first 48 h post-surgery, the 5 latter probably due to malignant edema. Of the 11 rats included in the study, 4 had a corticostriatal lesion, 3 a striatal lesion and 4 a hypothalamic lesion (Fig. 2, Supplementary Fig. S1). Body weight changes over time were similar for these 3 groups (Supplementary Fig. S2). Five animals received intracerebral hASC treatment (Fig. 2, Supplementary Fig. S1).

### Neurofunctional readouts

#### Staircase test

**Figure 3:**
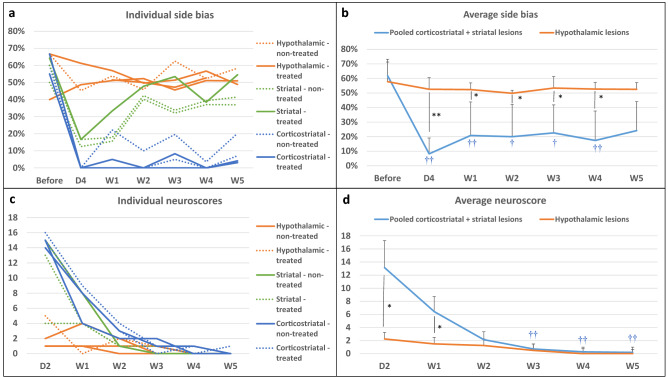
Neurofunctional readouts. (**a**) Individual side bias according to lesion subtype and treatment group (plain line: treated; dashed line: non-treated) in the first 5 weeks post-surgery. (**b**) Average side biases according to lesion subtype: pooled (corticostriatal + striatal) vs. hypothalamic lesions. (**c**) Individual neuroscores according to lesion subtype and treatment group (plain line: non-treated; dashed line: treated) in the first 5 weeks post-surgery. (**d**) Average neuroscores according to lesion subtype: pooled (corticostriatal + striatal) vs. hypothalamic lesions. Data are displayed as mean ± SD. *W* weeks, *D* days. *p < 0.05, **p < 0.01, (corticostriatal + striatal) vs. hypothalamic, Wilcoxon–Mann–Whitney test; ^†^p < 0.05, ^††^p < 0.01, D4 to W5 vs. before, Friedman test.

Figure 3a presents individual results for side bias according to lesion subtype and treatment group. Rats were slightly lateralized before surgery (side bias: 66% [56%; 66%]). Side bias in the hypothalamic-only lesion group was maintained around 50% (i.e., no difference between right and left paw performances) right after surgery and until the end of testing. In rats with striatal and corticostriatal lesions, side bias was severely increased in the first days after stroke (i.e., marked difference in favor of the ipsilateral paw). Rats with striatal lesions recovered nearly to the level of rats with hypothalamic lesions at W2, while rats with corticostriatal lesions remained severely impaired until W5 (Fig. 3a; W5: 6% [4%; 10%] for corticostriatal lesions *vs* 41% [39%; 48%] for striatal lesions and 51% [50%; 55%] for hypothalamic-only lesions). The changes in time in the number of pellets taken by the control non-affected paw was similar for rats with corticostriatal, striatal and hypothalamic lesions, thus suggesting that motivation to reach and eat pellets was the same for each time point, regardless of lesion subtype (Supplementary Fig. S2). Staircase performance remained low until W14 in rats with corticostriatal lesions (Supplementary Fig. S3). Intracerebral administration of hASCs did not negatively impact staircase test performance (Table 1).

**Table 1:** Neurofunctional and neuroimaging readouts according to treatment group.

Biomarkers		Time points		Treated (N = 5)	Non-treated (N = 6)	p
Neuroscores		Before treatment D2		11 ± 6	8 ± 7	0.4059
		Before treatment W1		5 ± 4	4 ± 3	0.7766
		After treatment W2		2 ± 1	2 ± 1	0.4493
		After treatment W3		1 ± 1	1 ± 1	1
		After treatment W4		0 ± 0	0 ± 0	1
		After treatment W5		1 ± 1	0 ± 0	1
Side bias (%)		Before stroke		58% ± 5%	58% ± 11%	0.576
		Before treatment D4		17% ± 20%	28% ± 27%	0.7787
		Before treatment W1		26% ± 18%	30% ± 27%	1
		After treatment W2		30% ± 23%	31% ± 25%	1
		After treatment W3		32% ± 19%	33% ± 25%	0.9307
		After treatment W4		27% ± 24%	33% ± 26%	0.9266
		After treatment W5		35% ± 22%	34% ± 24%	0.9307
Lesion size (mm^3^)		Before treatment D4		129 ± 120	77 ± 92	0.5368
		After treatment W5		61 ± 78	41 ± 56	0.6473
DTI	FA	Contra	Before tt D4	0.28 ± 0.03	0.27 ± 0.03	0.5368
			After tt W5	0.26 ± 0.05	0.26 ± 0.02	0.9307
		Ipsi	Before tt D4	0.19 ± 0.03	0.22 ± 0.05	0.2468
			After tt W5	0.38 ± 0.02	0.36 ± 0.02	0.2468
	MD	Contra	Before tt D4	0.76 ± 0.01	0.78 ± 0.04	0.5704
			After tt W5	0.77 ± 0.01	0.75 ± 0.01*	0.0365
		Ipsi	Before tt D4	0.71 ± 0.06	0.75 ± 0.07	0.358
			After tt W5	0.86 ± 0.17	0.86 ± 0.09	0.9307
	AD	Contra	Before tt D4	0.97 ± 0.10	1.02 ± 0.06	0.407
			After tt W5	0.98 ± 0.04	0.98 ± 0.03	0.8541
		Ipsi	Before tt D4	0.91 ± 0.13	0.92 ± 0.10	0.7144
			After tt W5	1.33 ± 0.19	1.24 ± 0.15	0.4286
	RD	Contra	Before tt D4	0.65 ± 0.01	0.66 ± 0.03	0.5778
			After tt W5	0.64 ± 0.04	0.65 ± 0.02	1
		Ipsi	Before tt D4	0.63 ± 0.05	0.65 ± 0.04	0.583
			After tt W5	0.72 ± 0.12	0.69 ± 0.09	0.9269

*Contra* contralateral, *D* days, *Ipsi* ipsilateral, *tt* treatment, *W* Weeks, *FA* fractional anisotropy, *MD* mean diffusivity, *AD* axial diffusivity, *RD* radial diffusivity.

p-values are given for Wilcoxon–Mann–Whitney test, *p < 0.05.

Because hypothalamic lesion did not have a neurofunctional impact on the staircase test, rats with hypothalamic-only lesions were assimilated to sham-like animals, while rats with striatal and corticostriatal lesions were considered as tMCAO rats and pooled for performing statistical analysis between 2 groups (N = 7 (corticostriatal + striatal) lesions *vs* N = 4 hypothalamic-only lesions). There was no difference in side bias across time in the hypothalamic-only lesion group (Friedman test—p = 0.8291), contrary to the pooled (corticostriatal + striatal) lesion group (Friedman test—p = 7.266e−05). Post-surgery side biases were statistically lower from pre-surgery ones, except at W5 (post-hoc Conover test—D4: p = 0.0002; W1: 0.0090; W2: 0.0249; W3: 0.0357; W4: 0.0080; W5: 0.1154). Side bias was statistically lower in pooled (corticostriatal + striatal) lesion group than in hypothalamic-only lesion group at all time-points, except before surgery (Fig. 3b; Wilcoxon–Mann–Whitney test—Before surgery: p = 1; D4: p = 0.0088; W1: p = 0.0105; W2: p = 0.0171; W3: p = 0.0424; W4: p = 0.0100; W5: 0.0424).

#### Neuroscores

Figure 3c,d present individual and averaged neuroscores according to lesion subtype and treatment group. Intracerebral administration of hASCs did not aggravate neuroscores (Table 1). At D2, neuroscores were in the same order of magnitude in rats with striatal lesion (13 [9; 14]) and rats with corticostriatal lesion (15 [15; 15]), while rats with hypothalamic-only lesion had much lower neuroscores (2 [1; 3]) (Fig. 3c), thus confirming our previous observations with regard to rats with hypothalamic-only lesions behaving as sham-like animals. There was a significant difference in neuroscores across time in the hypothalamic-only lesion group (Friedman test—p = 0.02727); however, none of the Conover post-hoc test were significant. In the pooled (corticostriatal + striatal) lesion group, the difference in neuroscores across time was statistically different (Friedman test—p = 4.897e−06), with W3, W4 and W5 neuroscores that were statistically lower than D2 neuroscores (Conover post-hoc test—W1: p = 0.3998; W2: 0.0955; W3: 0.0055; W4: 0.0015; W5: 0.0011). There was a statistically significant difference between the 2 groups at D2 that was attenuated but maintained at W1 (Fig. 3d; Wilcoxon–Mann–Whitney test—D2: p = 0.0168; W1: p = 0.0188). Starting at W2, neuroscores were no longer significantly different between groups, as all neuroscores had reached sham-like levels (Fig. 3d; Wilcoxon–Mann–Whitney test—W2: p = 0.3276; W3: p = 0.754; W4: p = 0.3241; W5: p = 0.5708).

### Imaging readouts

#### Brain lesions

Figure 2b,c show individual and average lesion sizes according to lesion subtype and treatment group. As expected, at D4, there was a gradient in lesion size according to subtype (D4: hypothalamic-only: 16 [5; 30] < striatal: 42 [28; 71] < corticostriatal: 227 [197; 249] mm^3^ and W5: hypothalamic-only: 2 [0; 5] < striatal: 8 [6; 9] < corticostriatal: 127 [119; 138] mm^3^). Corticostriatal lesion volumes remained stable after W5 (Supplementary Fig. S3; W9: 124 [111; 141] mm^3^). Intracerebral administration of hASCs did not impact lesions sizes (Table 1). Lesion shrinkage between D4 and W5 was statistically significant in pooled (corticostriatal + striatal) lesion group (Friedman test—p = 0.0081) but not in hypothalamic-only lesion group (Friedman test—p = 0.0832). Lesion size was statistically larger in pooled (corticostriatal + striatal) lesion group than in hypothalamic-only lesion group at both time-points (Fig. 2c; Wilcoxon–Mann–Whitney test—D4: p = 0.0424 and W5: 0.0293).

#### Microstructural alterations

**Figure 4:**
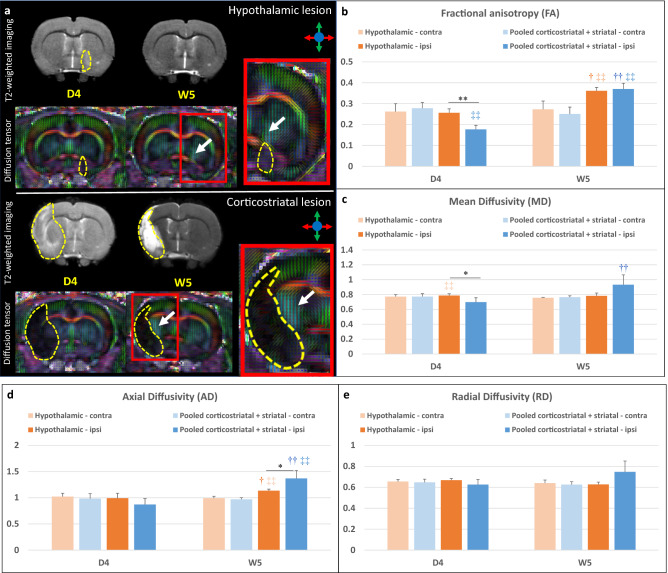
DTI readouts. (**a**) Example of T2-weighted imaging and color-coded fractional anisotropy for two individual rats: one with a hypothalamic lesion and one with a corticostriatal lesion (dotted yellow line). Only the central slice is presented. The internal capsule is pointed out by the white arrow. (**b–e**) Average DTI metrics (respectively FA, MD, AD and RD) are presented according to lesion subtype: pooled (corticostriatal + striatal) vs. hypothalamic lesions at day 4 (D4) and week 5 (W5) post-surgery. *FA* fractional anisotropy, *MD* mean diffusivity, *AD* axial diffusivity, *RD* radial diffusivity. *p < 0.05, **p < 0.01, (corticostriatal + striatal) vs. hypothalamic, Wilcoxon–Mann–Whitney test; ^†^p < 0.05, ^††^p < 0.01, W5 vs. D4, Friedman test; ^‡^p < 0.05, ^‡‡^p < 0.01, ipsilateral (ipsi) vs. contralateral (contra) side, Friedman test.

The internal capsule appeared as a region characterized by a high fractional anisotropy (FA) value localized between the lateral ventricle and the caudate putamen (Fig. 4a, white arrows). Supplementary Figure S4 and Fig. 4b–e present individual and average DTI metrics (MD: mean diffusivity, AD: axial diffusivity and RD: radial diffusivity) according to lesion subtype and treatment group. DTI metrics were not impacted by intracerebral administration of hASCs (Table 1). DTI metrics did not change over time in the contralateral internal capsule in hypothalamic-only lesion group (Friedman test—FA: p = 1; MD: p = 0.3173; AD: 0.3173; RD: 0.5637) and in pooled (corticostriatal + striatal) lesion group (Friedman test—FA: p = 0.2568; MD: 0.4142; AD: 0.2568; RD: 1). Also, there was no significant difference between these 2 groups in the contralateral internal capsule at D4 (Wilcoxon–Mann–Whitney test—FA: p = 0.5273; MD: p = 0.769; AD: 0.5044; RD: 0.6311) and W5 (Wilcoxon–Mann–Whitney test—FA: p = 0.3152; MD: p = 0.3028; AD: 0.1829; RD: 0.7035) (Supplementary Fig. S4, Fig. 4b–e). In the ipsilesional internal capsule, FA and MD were significantly decreased at D4 in the pooled (corticostriatal + striatal) lesion group compared to the hypothalamic-only lesion group (Fig. 4b,c; Wilcoxon–Mann–Whitney test—FA: p = 0.0060 and MD: p = 0.0363, represented by *). FA and AD were significantly increased in the ipsilesional and internal capsule at W5 vs. D4 (represented by †) in both hypothalamic-only lesion group (Friedman test—FA: p = 0.0455 and AD: p = 0.0455) and (corticostriatal + striatal) lesion group (Friedman test—FA: p = AD: p = 0.0081). MD was significantly increased at W5 vs. D4 in the (corticostriatal + striatal) lesion group only (Friedman test—p = 0.0081). FA was significantly decreased in the ipsilesional vs. contralesional internal capsule (represented by ‡‡) at D4 in (corticostriatal + striatal) lesion group (Friedman test—p = 0.0081). At W5, it was significantly increased in the ipsilesional vs. contralesional internal capsule both in hypothalamic-only lesion group (Friedman test—p = 0.0455) and in (corticostriatal + striatal) lesion group (Friedman test—p = 0.0081). AD was also significantly increased in the ipsilesional vs. contralesional internal capsule both in hypothalamic-only lesion group (Friedman test—p = 0.0455) and (corticostriatal + striatal) lesion group (Friedman test—p = 0.0081) at W5. In rats with a corticostriatal lesion, FA remained relatively stable in the ipsilesional internal capsule while MD, AD and radial diffusivity (RD) continued to increase at W9 (Supplementary Fig. S3).

### Post-mortem analysis

An increase in the internal capsule thickness was clearly seen in the ipsilesional side (Supplementary Fig. S5). The cellularity was enhanced in the ipsilesional internal capsule as seen by the increased number of nucleus compared to the contralateral side, which suggest the presence of inflammatory cells. CD68-positive cells (for macrophages) were not seen in the internal capsule and mainly found in the perilesional area. A high density of GFAP-positive cells was also observed in the perilesional area, corresponding to the glial scar. GFAP is expressed constitutively in astrocytes as seen in the contraleral internal capsule with a typical ramified morphology. In the ipsilesional internal capsule, GFAP staining was present in association with numerous nucleus suggesting astrogliosis. In addition, astrocytes presented a reactive phenotype compared to the control side.

### Therapeutic trial design

#### Correlation between imaging and neurofunctional readouts

**Figure 5:**
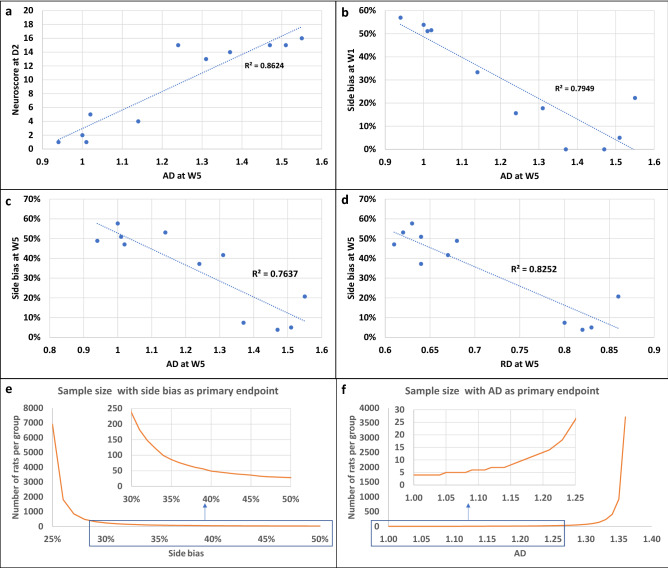
Correlations between neurofunctional and imaging outcomes and sample size calculation. (**a–d**) Main correlations between neurofunctional and imaging readouts. (**a**) Correlation between neuroscores at D2 and AD at D5 (Pearson correlation test, p = 3.65e−05). (**b**) Correlation between side bias at W1 and AD at W5 (Pearson correlation test, p = 0.00022). (**c**) Correlation between side bias at W5 and AD at W5 (Pearson correlation test, p = 0.00034). (**d**) Correlation between side bias at W1 and RD at W5 (Pearson correlation test, p = 6.543e−05). (**e,f**) Sample size calculation for future pre-clinical therapeutic trial. (**e**) Side bias as primary endpoint. The x-axis represents the hypothesized value of side bias in the treatment group and the y-axis represents the corresponding number of rats per group. (**f**) AD as primary endpoint. The x-axis represents the hypothesized value of AD in the treatment group and the y-axis represents the corresponding number of rats per group. *AD* axial diffusivity, *RD* radial diffusivity.

Several correlations were observed between imaging and neurofunctional readouts. Neuroscores at D1 and side bias at W5 were linearly related to lesion size at D4 (Supplementary Fig. S6). Figure 5 shows the most significant correlations between side bias and DTI metrics. AD at W5 correlated with neuroscore at day 2 (Fig. 5a; Pearson’s correlation—p = 3.65e−05) and with side bias at W1 (Fig. 5b; Pearson’s correlation—p = 0.0002) and W5 (Fig. 5c; Pearson’s correlation—p = 0.0003). Hence this parameter seemed the most relevant for detecting a treatment effect.

#### Sample size calculation

To assist in the design of future therapeutic trials, we performed calculations using our data to determine sample size in order to detect a deficit in treated compared to non-treated tMCAO rats at W5 after stroke. We assumed that the tMCAO group included corticostriatal and striatal lesions and excluded hypothalamic-only lesions. The primary endpoints were side bias (Fig. 5e) and the DTI metric AD (Fig. 5f). For the staircase test, we assumed a side bias value of 24% ± 20% in the non-treated tMCAO group (i.e., mean ± SD from the pooled (corticostriatal + striatal) lesion group of the current study at W5). The side bias in the treated group was then varied from 25 to 50% (corresponding to hypothalamic-only lesion levels at W5) by steps of 1% and sample size was calculated for each side bias (Fig. 5e). For side bias increasing from 24% to 32% (30% improvement in side bias), sample size needs to be 147 rats per group in order to detect a significant difference between groups. For side bias increasing from 24% to 36% (50% improvement in side bias, i.e. to reach the level of rats with striatal lesions), sample size needs to be 76 rats per group. For DTI metrics, we assumed an AD value of 1.37 ± 0.15 in the non-treated group (i.e., mean ± SD from the pooled (corticostriatal + striatal) lesion group of the current study at W5). AD in the treated group was then varied from 1.36 to 0.99 (corresponding to contralateral hypothalamic-only lesion levels at W5) by steps of 0.01 and sample size was calculated for each AD value (Fig. 5F). For an AD value decreasing from 1.37 to 1.23 (10% improvement in AD value, reaching the level of rats with striatal lesions), sample size needs to be 18 rats per group. For an AD value decreasing from 1.37 to 1.16 (15% improvement in AD value, i.e. to reach an intermediate value between rats with striatal and hypothalamic lesions), sample size needs to be 9 rats per group only.

## Discussion

**Figure 6:**
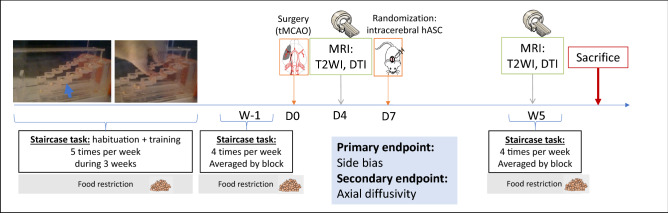
Design of preclinical therapeutic trial. The study design involves 2 staircase tasks (one before tMCAO surgery and one 5 weeks after, i.e. 4 weeks post-treatment) and 2 MRIs with T2-weighted imaging and DTI sequences (one at D4 post-surgery and one at 5 weeks after tMCAO surgery, i.e. 4 weeks post-treatment).

We here report an observational, descriptive study aiming to design a larger-scale therapeutic trial to evaluate intracerebral administration of hASCs in the chronic stage of ischemic stroke. Figure 6 summarizes the final protocol design.

Although such preclinical trials have already been published in the literature^29–31^, several methodological aspects, including choice of biomarkers, need to be considered to produce robust data that may be translated to the clinical realm. We chose to model ischemic stroke in rats using transient occlusion of the middle cerebral artery with an intraluminal thread. This model produces cerebral damage with a variety of lesion sizes and anatomical locations (hypothalamus, striatum and cortex)^28^, as is common in ischemic stroke patients^15^. Because lesion location, in addition to lesion size, is a main determinant of functional outcome, we reported our results according to these 3 lesion subtypes.

Long-term neurofunctional tests remain a challenge due to the quick compensation of rodents and the difficulty to differentiate adaptive strategy from motor improvement^13,32,33^. The test must be quantitative, allow repetition and it must reveal long term and stable deficits with enough sensitivity to show an improvement in treated *vs* non-treated subjects^34,35^. The staircase test, a skilled reaching task that assesses forelimb function, fulfills these criteria for the long-term evaluation of motor recovery in stroke-induced rats^36,37^. However, the optimal frequency of testing, the timescale and side bias modifications in time still remained to be determined^38–41^. Our results confirm that the staircase test is an appropriate neurofunctional biomarker for the long-term evaluation of rats with corticostriatal and striatal lesions. To the best of our knowledge, this is the first study to report detailed changes in side bias over time according to lesion subtype using the tMCAO intraluminal thread model. Because of the heterogeneity between striatal and corticostriatal lesions, the number of subjects per group to detect a side bias difference in a stem-cell trial is relatively high (76–147 rats per group). This is not unfeasible but necessitates a multicenter design, probably including at least 5 centers, as in Ref.^42^. Because animals with corticostriatal and striatal lesions present different sensorimotor deficits and recovery, complementary analysis may need to be performed in these subgroups, provided that the number of included animals provides sufficient statistical power. Alternatively, pre-treatment T2WI may be used to include rats with corticostriatal lesions only, in order to reduce variability and hence sample size. This might be relevant for translational research, as half of stroke patients experience persistent loss of upper-limb function in the chronic stage^16^.

One limitation of the staircase test is that it requires intensive training and is prone to large exclusion rates of low-learner rats^37^. In addition, implementation and analysis are quite strenuous and time-consuming. Hence we aimed at simplifying the neurofunctional follow-up. As side bias remained stable in all groups 2 weeks after tMCAO, a single week of testing (with block averaging) may be sufficient to assess stem-cell treatment effects. We suggest choosing W5, because we found no further spontaneous improvement after this stage, while a follow-up of at least one month after treatment is usually recommended for stem-cell studies^34,43^. Another limitation of the staircase test is the necessity of food restriction to favor motivation. Some recent reports have shown a neuroprotective effect of food restriction^44,45^ so food restriction may induce a bias when evaluating stroke recovery. Nevertheless, both body weights and motivation was found to be similar at all time-points for the 3 groups of lesion subtypes thus suggesting that that bias did not differently impact animals from the different groups.

Advanced neuroimaging modalities such as DTI are commonly used in clinical stroke research as complementary outcome measures to neurofunctional evaluation^19^. Axial diffusivity appeared to be the most relevant parameter. We observed a decrease (although not significant) in AD in the ipsilesional internal capsule in the acute stage of ischemic stroke and a significant increase in the chronic stage. There was also a trend toward an increase in RD in the chronic stage in the ipsilesional internal capsule. This is consistent with acute axonal damage followed by chronic axon and myelin damage^46^. Post-mortem immunohistological data also suggested the potential sensitivity of DTI metrics to glial activation as already reported in the literature^47^. The hypothesis to test in a stem cell trial would be that CST microstructural remodeling and immunomodulation by treatment ‘normalizes’ the AD value. Because DTI metrics are quite consistent over time and across animals, the number of subjects per group if this metric is used as a primary endpoint (N = 9–18 according to the expected size effect) would be compatible with a single-center exploratory study. However, such a study would be underpowered to evaluate side bias at the same time, and should therefore be considered preliminary.

We did not observe any impact of intracerebral administration on neurofunctional and imaging readouts. This is important to report as the main drawback of this route of administration is its invasiveness. We thus confirm the safety of the procedure. On the other hand, there was no trend toward an improvement of any of the endpoints evaluated in the group of rats that were injected with hASC compared to those who were not. Although the study was not powered to detect such an effect, this suggests that cell therapy regimen may benefit from being optimized before proceeding to the larger-scale preclinical trial. In particular, perilesional rather than intra-striatal administration of hASC may provide better efficacy.

The main limitation of the present study was the small number of animals that were included. This was due in part to a mortality rate that was higher than expected, probably due to complications such as hemorrhagic transformation and malignant edema that are difficult to anticipate. Exclusion rates are rarely reported in stroke research, which actually represents one of the methodological flaw of preclinical studies. Basalay et al. recently reported a 25% exclusion rate at 24 h post-surgery due to the combination of mortality rate and hemorrhagic transformations in a bicentre international study using the same tMCAO model in rats^48^. For long-term studies, the mortality rate is increased as seen in the current study, where the mortality post-surgery exceeded our a priori hypothesis: this is a further element to be taken into account when designing a therapeutic trial to reach the adequate statistical power. Nevertheless, the study was designed as an observational study, results are shown for individual animals and statistical analysis was performed between pooled (corticostriatal and striatal lesions) lesions and sham-like (hypothalamic lesions) groups only. We therefore believe these results are robust and that they are of interest to the stroke community by addressing the need to standardize preclinical stem-cell trials and design high-quality studies. The next step will be to validate the selected neurofunctional and imaging readouts in a larger exploratory study.

## Conclusion

This study determined the optimal neurofunctional and imaging readouts for the follow-up of rats in the chronic stage of ischemic stroke, the relevant timescale, and adequate sample size to evaluate the therapeutic effects of intracerebral administration of hASCs, in line with international recommendations^34^. We conclude that an exploratory preclinical trial based on both readouts would be feasible only in the framework of a multicenter trial, which in turn would necessitate appropriate funding and/or an industrial partnership. Such rigorous approaches are paramount for the successful translation of preclinical stem-cell research for the benefit of stroke patients.

## Methods

### Animals and ethics statement

All experimental procedures involving animals and their care conformed to European regulations for animal use (APAFIS agreement number: APAFIS#4688-2016032514131943). This study was approved by our institutional ethic committee “Comité d’éthique pour l’Expérimentation Animale Neurosciences Lyon” (CELYNE-CNREEA number: C2EA-42). The rats were housed three to four per cage (except in the first 2 days post-surgery where they were housed one per cage) in a temperature and humidity-controlled environment (21.2 ± 3 °C), on 12:12 h light–dark cycle, having free access to tap water and standard diet except during neurofunctional testing when they were put under food restriction for motivation (see details below). Rats were housed, regardless of type of lesion or treatment, in a standard Plexiglas box covered with mulch and enriched with red-colored cylindrical plastic tubes. Male Sprague Dawley OFA rats (Crl:OFA(SD), Charles River, France) aged 6–8 weeks were used, with a mean weight of 199 ± 13 g at the start of the experiment, i.e. when they started habituation and training for the staircase task. Rats body weight was 268 ± 17 g at D0 when they underwent surgery.

### Sample size, inclusion criteria and blinding

The RIGOR guidelines were used to design the study^49^. Data were reported according to ARRIVE (Animal Research: Reporting of In Vivo Experiments) guidelines^23^. Inclusion criteria were: lesion on D4 T2-weighted imaging, regardless of size and location, and complete neurofunctional and imaging follow-up. No formal sample size was calculated for this observational, descriptive study. We aimed at including 15 rats within 1 year. We thus planned to enroll 25 animals, assuming a 20% exclusion rate for low-learners on the staircase test^37^ and 25% exclusion for the tMCAO model due either to mortality or to absence of lesion on T2-weighted imaging at D4^48^. All data were anonymized and analyses were performed blindly. For the staircase test analysis, treatment group allocation was concealed by filming the rats from the side so that rats that had undergone intracerebral administration could not be identified.

### Ischemic stroke model

The animal model of ischemic stroke was the previously described tMCAO model^50^. Rats were anesthetized with a mixture of isoflurane and ambient air (4% during induction and between 1 and 2% during surgery) (ISO-VET, Piramal Healthcare, Morpeth, UK). Analgesia was obtained with subcutaneous administration of buprenorphine (Buprecare, Axience) at 0.05 mg/kg, injected after anesthesia induction. Briefly, the model was performed by introducing the thread (Doccol corporation, USA) through the external carotid artery. The thread was kept in place for 60 min. Because we aimed at inducing variability in a limited number of subjects (in order to investigate lesion subtypes), the thread size was the same for all rats (0.39 mm) and not adapted to the rat weight as we usually do. In these conditions, hypothalamic lesions are seen in a non-negligible proportion of operated rats, while striatal and corticostriatal lesions are equally expected^51^. Definitive occlusion of the internal carotid artery and external carotid artery was performed. Temperature was controlled with a rectal probe throughout the surgical procedure with a heating pad set at 37 °C. The effectiveness of occlusion was checked by the presence of a lesion on D4 T2-weighted imaging.

### Stem-cells

Surgical residue was harvested according to French regulations and declared to the Research Ministry (DC no 2008162) following written informed consent from the patients. Human stromal vascular fraction (SVF) was isolated from lipoaspirate obtained from healthy volunteers undergoing liposuction. Adipose tissue was digested with collagenase (0.120 U/ml, Roche, Indianapolis, IN, USA) at 37 °C for 30 min and under constant shaking. Digestion was stopped by adding Dulbecco’s Modified Eagle’s Medium (DMEM with Glutamax^®^, Gibco^®^, Invitrogen, Carlsbad, CA, USA) containing 10% fetal calf serum (FCS, HyClone, Logan, UT, USA). Floating adipocytes were discarded and cells from the SVF were pelleted, rinsed with medium, centrifuged (300×*g* for 5 min at 20 °C) and incubated in an erythrocyte lysis buffer for 20 min at 37 °C. The cell suspension was centrifuged (300×*g* for 5 min, 20 °C) and cells were counted using the Trypan blue exclusion method. A total of 40,000 SVF cells/cm^2^ were plated and grown in proliferation medium containing DMEM (Gibco^®^, Life technologies), HAM-F12 l-Glutamine (Gibco^®^, Life Technologies, St Aubin, France) (v/v), 10% FCS (HyClone), 10 ng/ml basic fibroblast growth factor (FGF2, Miltenyi Biotec, Paris, France), 10 μg/ml Gentamicin and 100 IU/ml Penicillin (Panpharma, Fougères, France). The medium was changed three times a week until 80% confluence was reached. At subconfluency, cells were detached with Trypsin-0.01%-EDTA (Gibco^®^ (Invitrogen, Carlsbad, CA, USA) and centrifuged for 10 min at 300×*g* and amplified in subculture at 4000 cells/cm^2^ density during two passages.

#### Stem-cell administration

A subgroup of animals received clinical-grade human mesenchymal stem cells derived from human adipose tissue (hASCs, codecoh number AC 2019-3476). Coordinates for stereotaxic injection were calculated from D4 MRI to inject cells in the striatal part of the lesion. Rats were anesthetized with the same protocol as for stroke induction and placed in a stereotaxic frame (Stoelting, Chicago, IL, USA) with a mask delivering isoflurane during the procedure. After opening the skin and careful drilling of the chosen entry point, 500,000 hASCs were prepared in 10-µl medium solution and injected in 1 min through a 26-gauge needle (RN-type, NH-BIO) with a UltraMicropump III with Micro4Controller (World Precision Instruments, Friedberg, Germany). The needle was kept in place for 2 min before careful progressive removal. The control subgroup did not undergo intracerebral surgery, as we aimed at assessing the impact of intracerebral administration on neurofunctional and imaging readouts.

### Neurofunctional testing

#### Staircase test

Staircase testing was performed under restricted feeding to maintain body weight at 90% of normal (0.05-g/g of weight)^36,38^. Weight was checked every day during the restriction period and the quantity of diet given daily after the staircase experiment was adapted in case of weight loss. No diet restriction was imposed for 2 days before and 7 days after stroke to allow good recovery from surgery. After a period of habituation to the experimenter (1 week) and to the pellets (Dustless precision pellets, purified, 45 mg, Bio-Serv, Flemington), rats were placed for 10 min 5 days a week in the home-made staircase box^38,39^. Rats with sufficient training (pellet retrieval ≥ 6 pellets in 10 min) were selected before stroke induction. Unilateral stroke was performed to impair the dominant side when rats were lateralized (i.e., taking more pellets on one side than the other: side bias > 60%), otherwise they were operated on the right side. Rats were filmed during their task (Sony Xperia ZD compact) and analyses were made by a blinded observer after anonymization of the movies. The number of pellets retrieved per side using the paw only was evaluated for each test session. With this apparatus, only the ipsilateral paw can take the pellet on the ipsilateral side and vice-versa. Data were averaged by blocks of 4 tests to obtain 1 side-bias value per week. Side bias was used to evaluate neurofunctional deficit and was calculated as contra/(ipsi + contra) performance, with ‘ipsi’ corresponding to the brain-spared side and ‘contra’ to the brain ischemic side^36^. Staircase tests were carried out in the morning or in the afternoon in alternation.

#### Neuroscore

The neuroscore was a scale from 0 to 20 that included sensorimotor tasks: gait, limb placing, parachute reflex, lateral resistance, beam walk^52^. A higher score indicates a more severe deficit. The test was performed in the morning.

### MRI

For in vivo MRI, anesthesia was induced and maintained in the same way as during the surgery. The animals were placed in an MRI-compatible rats cradle. The respiratory rhythm was monitored by a pressure sensor linked to a monitoring system (ECG Trigger Unit HR V2.0, RAPID Biomedical, Rimpar, Germany). MRI acquisitions were performed on a horizontal 7T BRUKER Biospec MRI system (Bruker Biospin MRI GbmH Bruker, Germany) equipped with a set of gradients of 440 mT/m and controlled via Bruker ParaVision 5.1 workstation. A Bruker birdcage volume coil (inner diameter = 72 mm and outer diameter = 112 mm) was used for the signal transmission, and a Bruker single loop surface coil (25 mm diameter) was used for signal reception. Two sequences were used: anatomical T2-weighted imaging (T2WI) and diffusion tensor imaging (DTI). Supplementary Table S1 presents the acquisition parameters.

#### Images analyses

Bruker raw data were converted in Nifti format using the open source medical image converter Dicomifier (https://github.com/lamyj/dicomifier). For assessment of lesion size, T2WI data were analyzed blindly using ImageJ software (National Institute of Health, USA imagej.nih.gox/ij/) by manually contouring the lesion, the ipsilateral and the contralateral hemispheres. The DTI parametric maps (fractional anisotropy FA, mean diffusivity MD, axial diffusivity AD and radial diffusivity RD) thus directional color-coded fractional anisotropy maps were obtained, after motion correction between volumes based on a rigid registration, using FSL (FMRIB Software Library, The University of Oxford). Then, affine registration according to the FA map at W5 was applied to individual maps using FSL. The internal capsule was analyzed to evaluate the ipsilesional corticospinal tract disruption and remodeling in analogy to patient studies^19,53^. A manual region of interest was used to delineate the ipsilesional striatum, where the internal capsule is located. Then the internal capsule was automatically obtained by thresholding according to a FA value superior to 0.3. The contralateral ROI was obtained by mirroring the ipsilateral ROI.

### Immunohistochemistry (IHC)

Rat brains were coronally sectioned in 10 µm using a cryostat (LEICA, Microsystems). Tissue sections on slides were fixed with 4% PFA for 10 min at RT and rinsed in PBS buffer. Immunohistochemistry (IHC) staining were performed on Discovery XT Automate (Roche), with the DAB Map Detection Kit (RUO), DISCOVERY (Cat#05266360001, Roche). Sections were subjected to heat-mediated antigen retrieval with Tris–EDTA Buffer (pH8) for 12 min at 95 °C. Incubation with primary antibody GFAP (Cat#2334, Dako) at 1/1000 was performed for 1 h at 37 °C. Tissues sections were incubated with Biotinylated Goat anti-rabbit IgG secondary antibody (Cat#BA1000, Vector) for 30 min at 37 °C. Sections were counterstained with hematoxylin. Slides were scanned on AxioScan Z1 (Zeiss) with a 20X objective.

### Statistical analysis

Statistical analysis was performed with R for Mac (The R foundation for statistical Computing). Data are given as median [25%; 75%] interquartile unless specified otherwise. Because the residual normality hypothesis was not verified, for longitudinal data, differences between time-points were evaluated with Friedman test followed by Conover post hoc test with p-value adjustment according to Holm method. Differences between two groups at a given time point were evaluated with a two-sided Wilcoxon–Mann–Whitney tests. The Pearson correlation test was used for correlation analysis. A p-value inferior to 0.05 was considered significant. Sample size calculation were made with G*Power 3.9.11.2 for a power of 0.8, an alpha error of 0.05 and 2-sided Wilcoxon–Mann–Whitney tests for two groups using the data obtained in the study at week 5 post-stroke (as further specified in the “Results” section).

## Supplementary Information

Supplementary Information.

## Data Availability

The processed data required to reproduce these findings and perform the statistical analyses are available to download at the figshare repository—https://figshare.com (https://figshare.com/s/15af2a099076389d2a5e).
