# Supplementary material for: Neurofunctional and neuroimaging readouts for designing a preclinical stem-cell therapy trial in experimental stroke
Source: Sci Rep. 2022 Mar 18;12:4700. doi: 10.1038/s41598-022-08713-z (PMC8933390; doi:10.1038/s41598-022-08713-z)
Supplement: Supplementary file 1 — Supplementary Information. [file 41598_2022_8713_MOESM1_ESM.docx]

**Neurofunctional and neuroimaging readouts for designing a preclinical stem-cell therapy trial in experimental stroke**

14 words **< 20 words**

Chloé Dumot^1, 2^, Chrystelle Po^3^, Lucille Capin^4^, Violaine Hubert^1^, Elodie Ong^1, 2^, Matthieu Chourrout^5^, Radu Bolbos^6^, Camille Amaz^7^, Céline Auxenfans^2,4^, Emmanuelle Canet-Soulas^1^, Claire Rome^8^, Fabien Chauveau^5,9^, Marlène Wiart^1,9,*^

1. Univ Lyon, CarMeN Laboratory, Inserm U1060, INRA U1397, INSA Lyon, Université Claude Bernard Lyon 1, Lyon, France
2. Hospices Civils de Lyon, Lyon, France
3. ICube, Université de Strasbourg, CNRS, UMR 7357, Strasbourg, France
4. Tissue and Cell Bank, HCL, Lyon, France
5. Univ Lyon 1, Lyon Neurosciences Research Center, CNRS UMR5292, Inserm U1028, Université Claude Bernard Lyon 1, Lyon, France
6. Cermep, Lyon, France
7. Clinical Investigation Center, CIC 1407, HCL, Louis Pradel Hospital, Lyon, France
8. Inserm, U1216, BP 170, 38042 Grenoble Cedex 9, France; Grenoble Institut des Neurosciences (GIN), Université Grenoble Alpes, 38000 Grenoble, France
9. CNRS, Lyon, France

*Corresponding author:

**Marlène WIART**

U1060 CARMEN-IRIS team

Groupement Hospitalier Est

Bâtiment B13, IHU OPERA

59 Boulevard Pinel

69500 BRON – France

[marlene.wiart@univ-lyon1.fr](file:///D:\Marlene\Papers\CDpaper%20-%20in%20progress\Septembre2021\marlene.wiart@univ-lyon1.fr)

**Twitter:** @MarleneWiart

| **MR imaging parameters** | **T2WI: 2D**  **RARE sequence**  **(RARE factor 8)** | **DWI: 2D diffusion-weighted echo planar imaging (EPI-multishot)** |
| --- | --- | --- |
| TE/TR (ms/ms) | 75/5,000 | 21/5,000 |
| Flip angle (degrees) | 180 | 90 |
| Number of averages | 2 | 2 |
| Bandwidth (kHz) | 35 | 300 |
| Field of view (mm^2^) | 35 x 35 | 35 x 35 |
| Slice thickness/interslice (mm) | 1/0 | 1/0 |
| Number of slices | 15 | 15 |
| Matrix size | 256 x 256 | 128 x 128 |
| *b* values (s/mm^2^) | N/A | 1000 |
| Number of shots | N/A | 4 |
| Number of directions | N/A | 30 |
| Diffusion gradient duration *δ* (ms) | N/A | 4.9 |
| Diffusion gradient separation *Δ* (ms) | N/A | 9.1 |
| Acquisition time (min, sec) | 5 min 20 sec | 11 min 40 sec |

TE: echo time; TR: repetition time; T2WI: T2-weighted MRI; DWI: diffusion-weighted MRI; N/A: not applicable.

**Supplementary Table 1- MR imaging sequences**

**Supplementary Figure legends**

**Supplementary Figure 1- CONSORT-like chart of the overall study.**

This figure presents the number of animals included at each step, the stratification according to lesion subtype and the allocation to treatment groups. CONSORT: Consolidated Standards of Reporting Trials.

**Supplementary Figure 2- Impact of food restriction on body weight and motivation. a.** Body weight. Food restriction was discontinued 2 days before tMCAO and during the first 7 days post-tMCAO, hence the increase in body weight around week 0 (W0). Apart from that, body weights remained stable in time; **b.** Number of pellets taken with the ipsilateral paw (i.e. non-affected side) expressed as a percentage of the number of pellets taken by the same paw the week before tMCAO. The number of pellets taken at D4 post-surgery was the same as the one taken before surgery, thus suggesting that the discontinuation of food restriction during the first week after surgery did not decrease the motivation to perform the test. W: week. tMCAO is performed at D0 of W0.

**Supplementary Fig 3- Follow-up of corticostriatal lesions until W14.**

**a.** Side bias; **b.** Lesion size; **c.** **FA:** Fractional anisotropy; **d.** **MD:** Mean diffusivity; **e.** **AD:** Axial diffusivity; **f.** **RD:** Radial diffusivity.

**Supplementary Figure 4- Microstructural alterations**

Individual DTI metrics are presented according to lesion subtype and treatment group (plain line: treated; dashed line: non-treated) at day 4 (D4) and week 5 (W5) post-surgery. **a.** FA ipsilateral side: corticostriatal and striatal lesions; **b.** FA contralateral side: corticostriatal and striatal lesions; **c.** MD ipsilateral side: corticostriatal and striatal lesions; **d.** MD contralateral side: corticostriatal and striatal lesions; **e.** AD ipsilateral side: corticostriatal and striatal lesions; **f.** AD contralateral side: corticostriatal and striatal lesions; **g.** RD ipsilateral side: corticostriatal and striatal lesions; **h.** RD contralateral side: corticostriatal and striatal lesions; **i.** FA ipsilateral side: hypothalamic lesions; **j.** FA contralateral side: hypothalamic lesion; **k.** MD ipsilateral side: hypothalamic lesions; **l.** MD contralateral side: hypothalamic lesion; **m.** AD ipsilateral side: hypothalamic lesions; **n.** AD contralateral side: hypothalamic lesion; **o.** RD ipsilateral side: hypothalamic lesions; **p.** RD contralateral side: hypothalamic lesion; FA: fractional anisotropy, MD: mean diffusivity, AD: axial diffusivity, RD: radial diffusivity.

**Supplementary Figure 5- Immunohistochemistry. a.** CD68 immunostaining; A1: contralateral internal capsule; A2: ipsilateral internal capsule; A3: perilesional area; **b.** GFAP immunostaining; B1: contralateral internal capsule; B2: ipsilateral internal capsule; B3: perilesional area.

**Supplementary Figure 6- Relationship between lesion size and neurofunctional outcome.**

**a.** Linear relationship between neuroscores at D2 and lesion size at D4; **b.** Linear relationship between side bias at W5 and lesion size at D4.


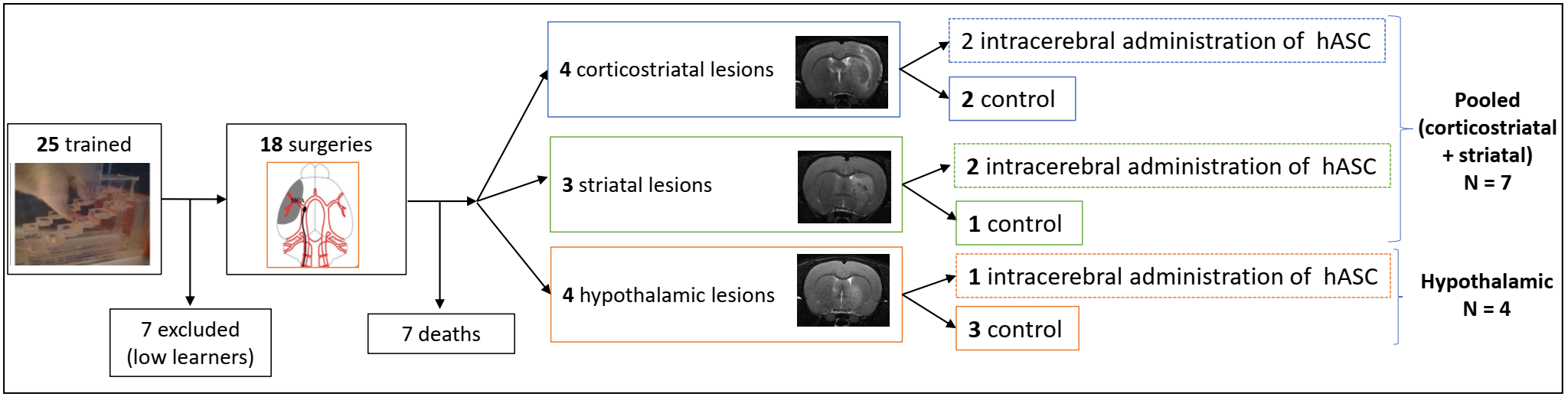


**Supplementary Figure 1- CONSORT-like chart of the overall study.** This figure presents the number of animals included at each step, the stratification according to lesion subtype and the allocation to treatment groups. CONSORT: Consolidated Standards of Reporting Trials.


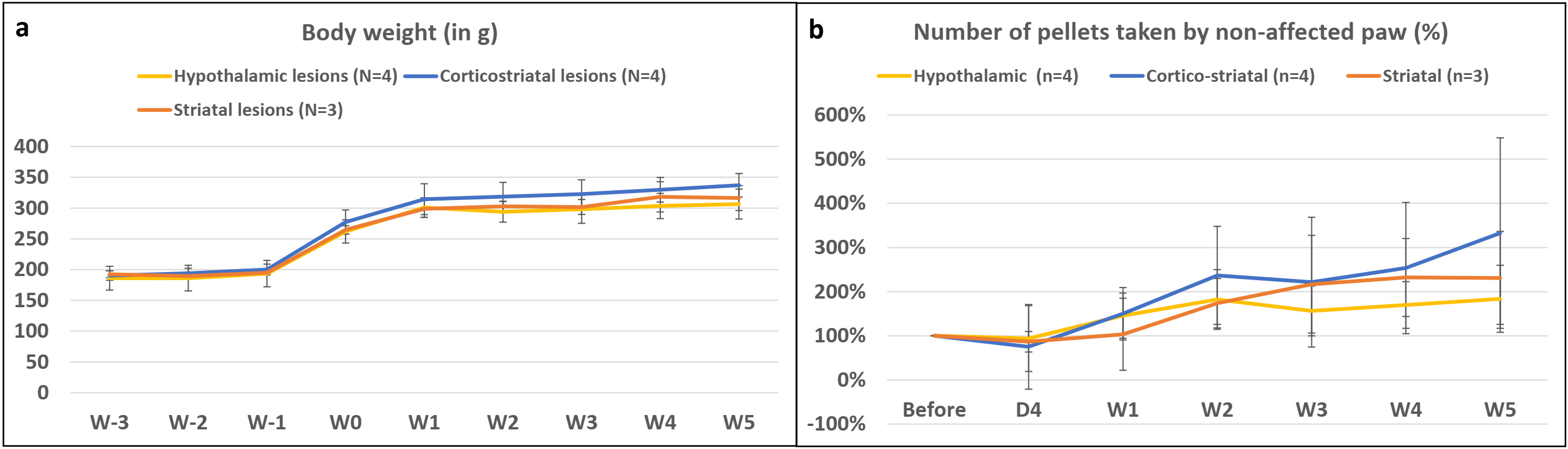
**Supplementary Figure 2- Impact of food restriction on body weight and motivation. a.** Body weight. Food restriction was discontinued 2 days before tMCAO and during the first 7 days post-tMCAO, hence the increase in body weight around week 0 (W0). Apart from that, body weights remained stable in time; **b.** Number of pellets taken with the ipsilateral paw (i.e. non-affected side) expressed as a percentage of the number of pellets taken by the same paw the week before tMCAO. The number of pellets taken at D4 post-surgery was the same as the one taken before surgery, thus suggesting that the discontinuation of food restriction during the first week after surgery did not decrease the motivation to perform the test. W: week. tMCAO is performed at D0 of W0.


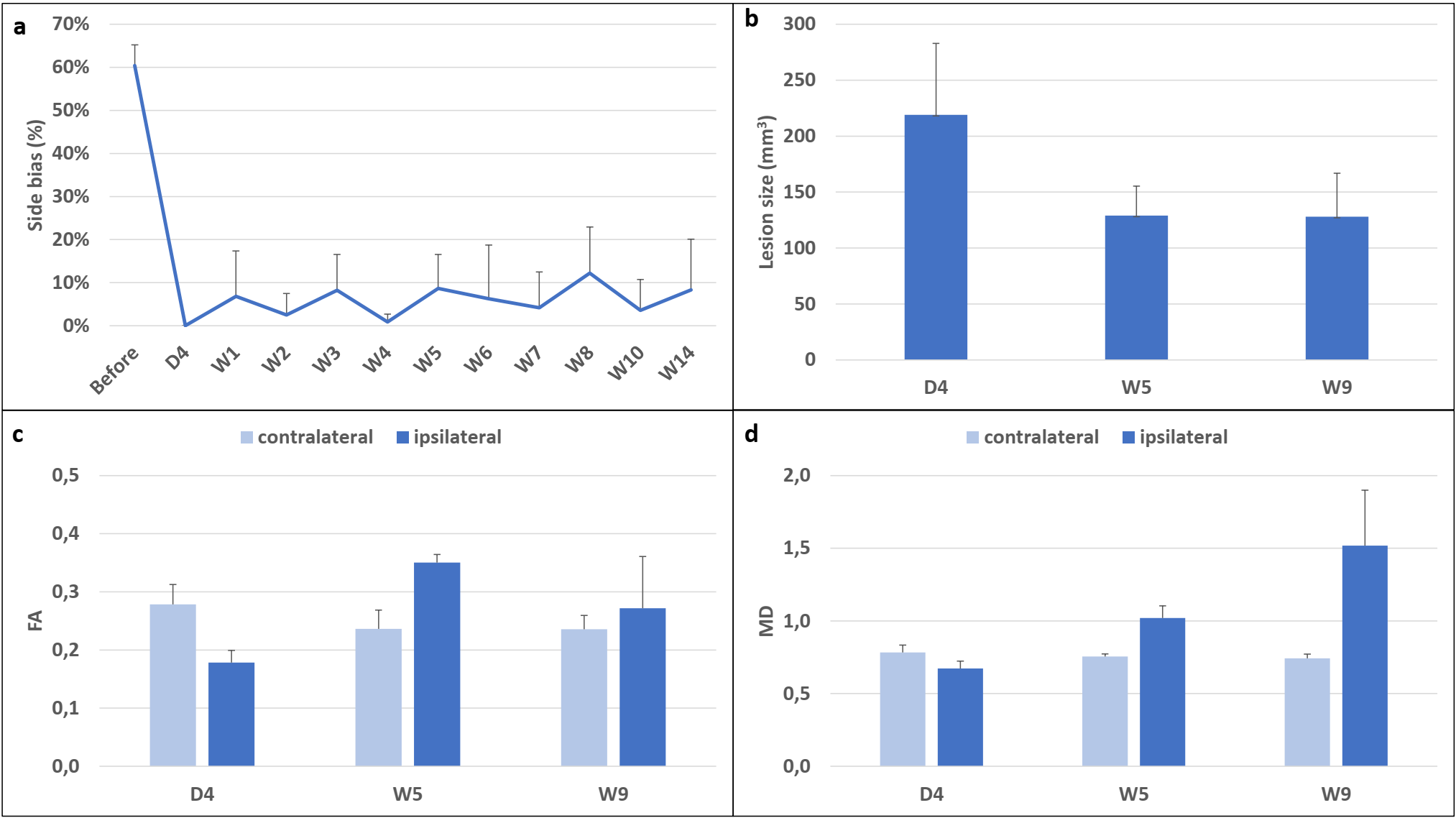


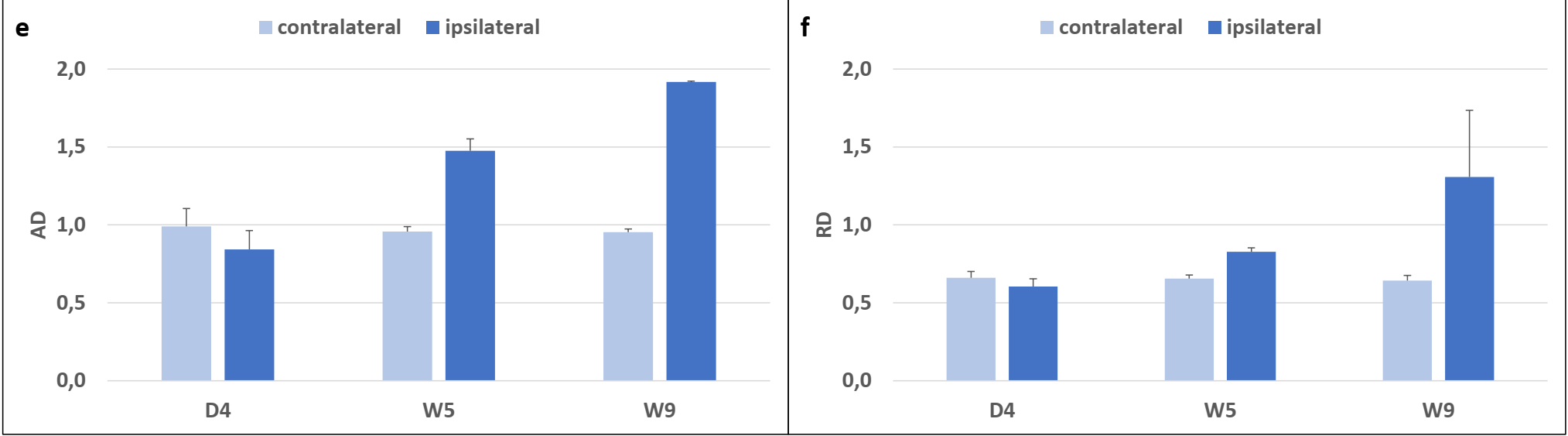


**Supplementary Fig 3- Follow-up of corticostriatal lesions until W14. a.** Side bias; **b.** Lesion size; **c.** **FA:** Fractional anisotropy; **d.** **MD:** Mean diffusivity; **e.** **AD:** Axial diffusivity; **f.** **RD:** Radial diffusivity.


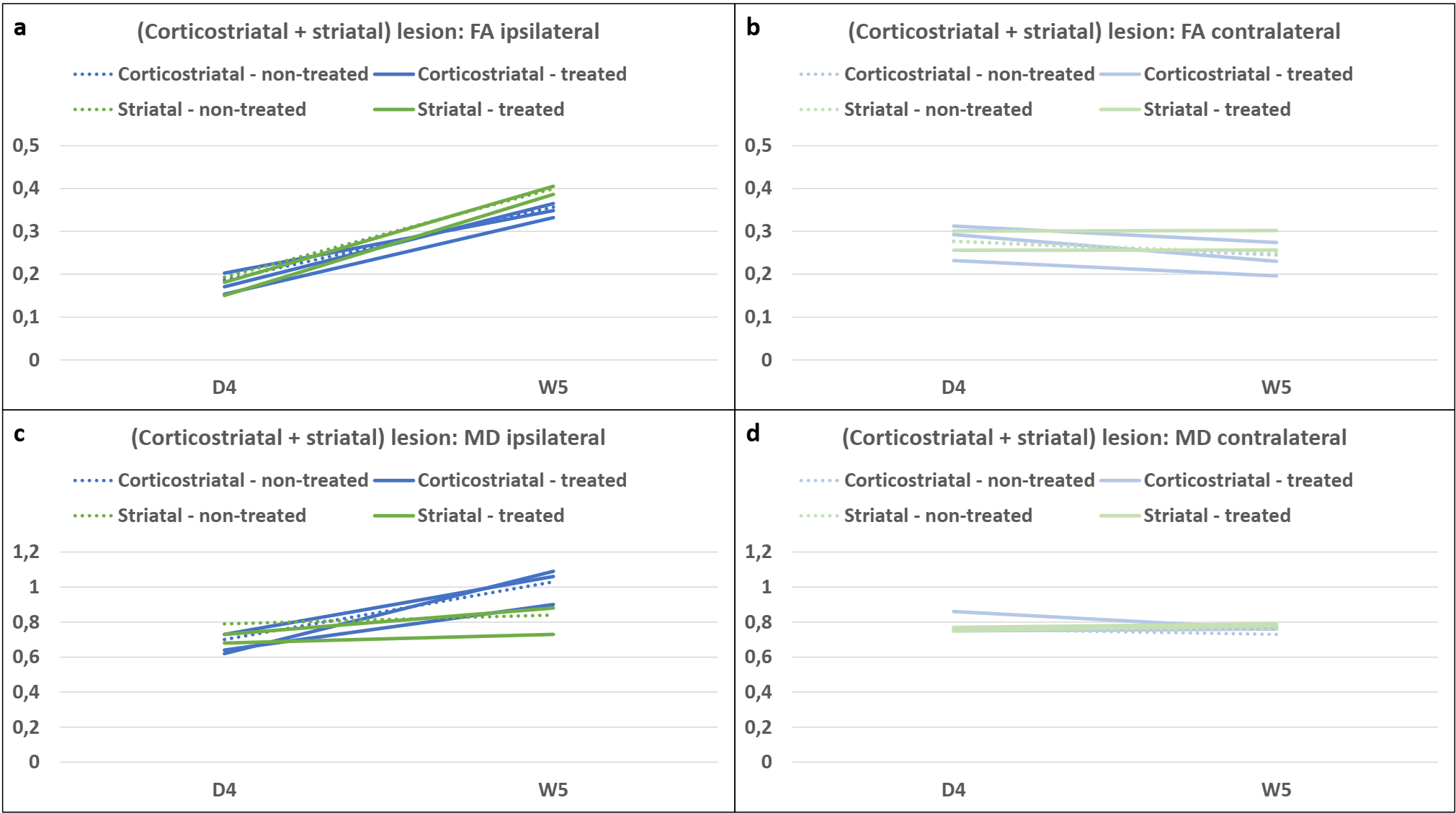

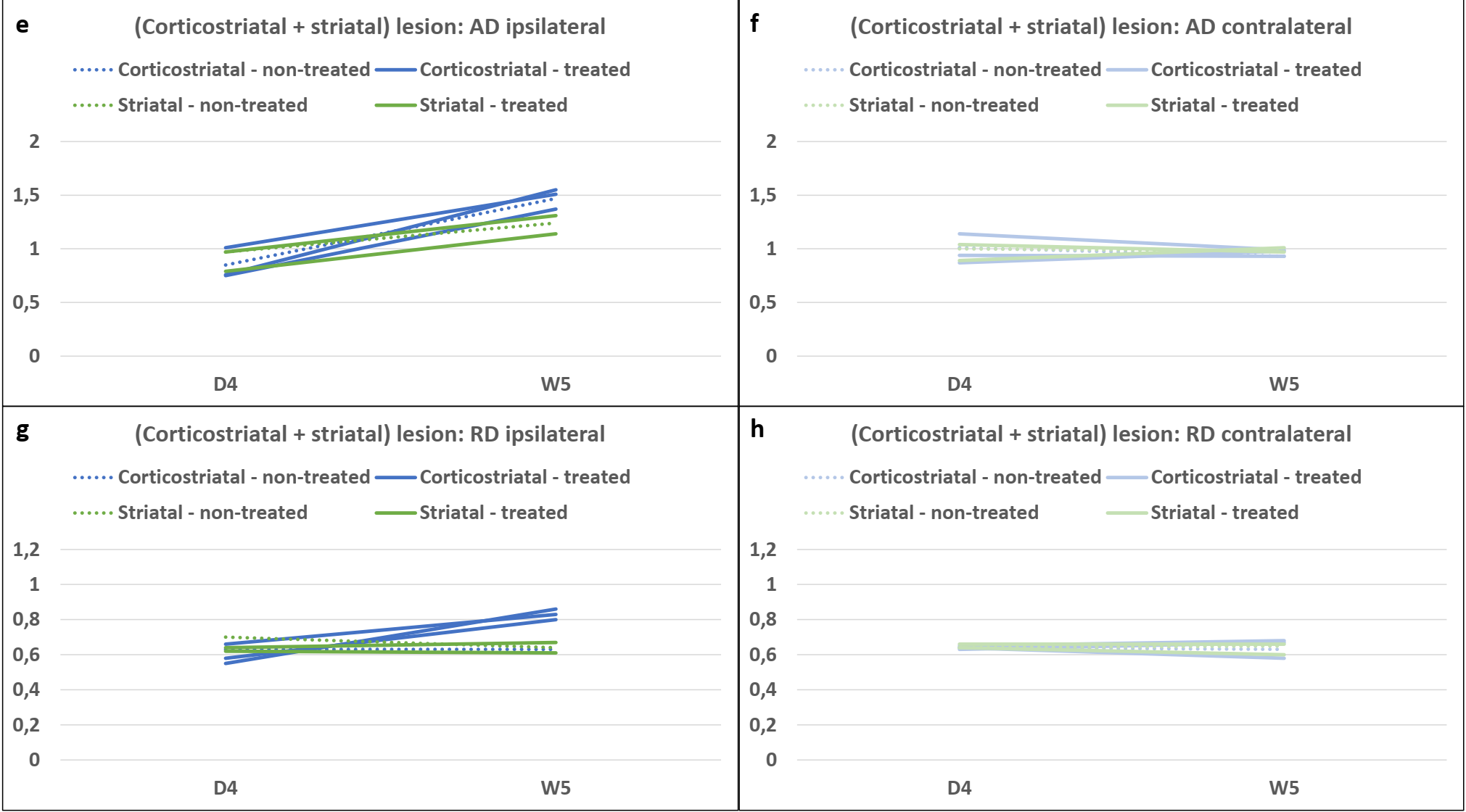

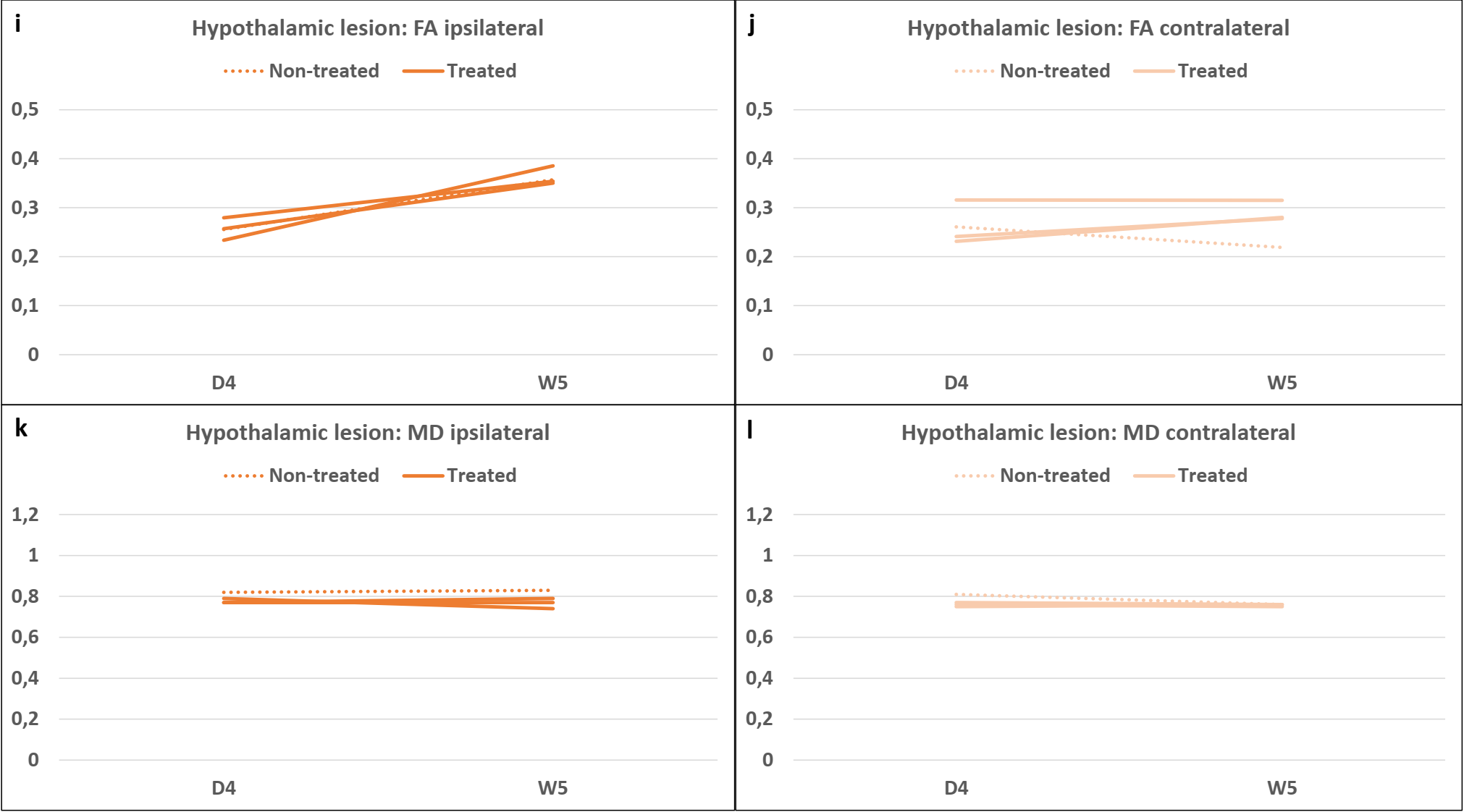

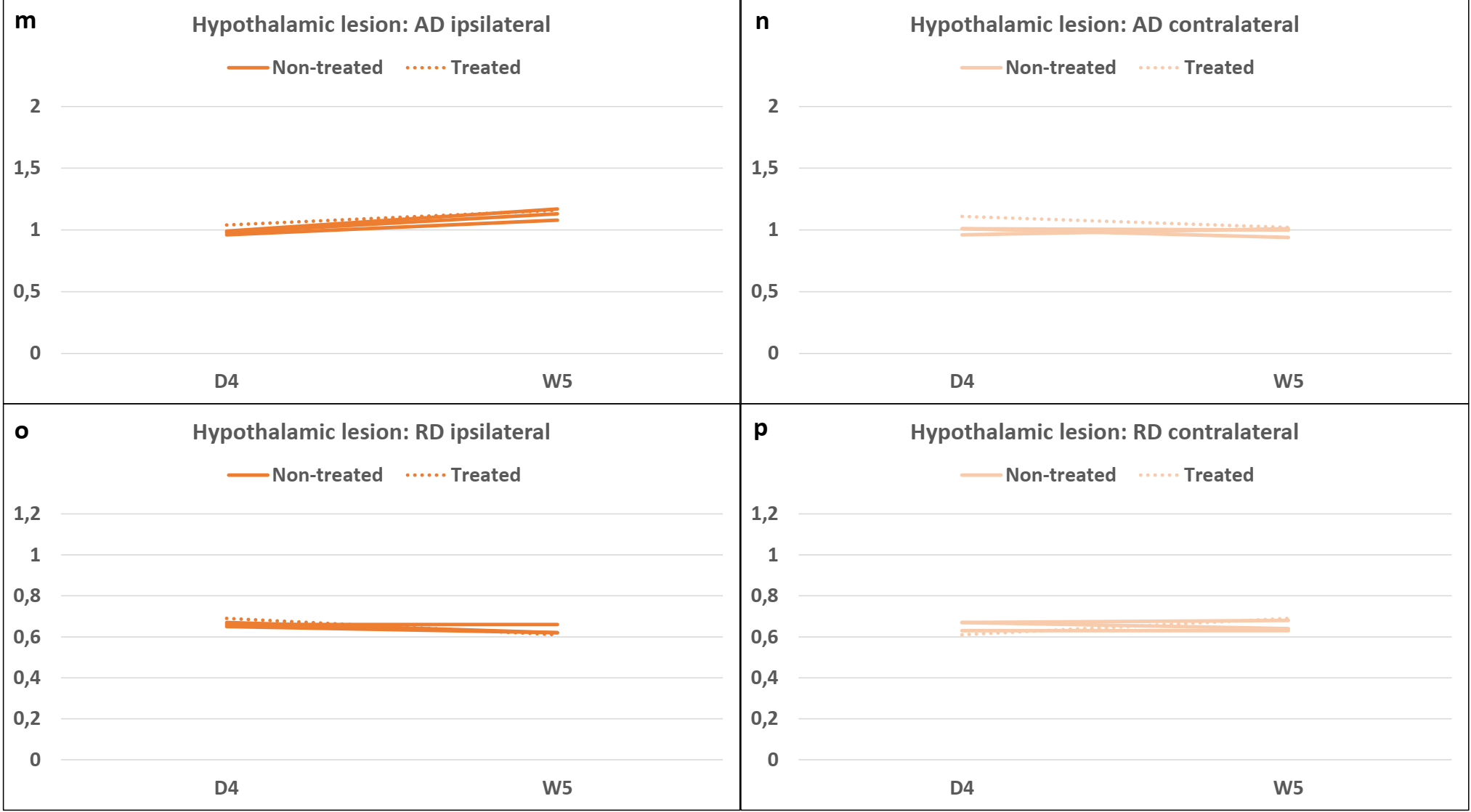


**Supplementary Figure 4- Microstructural alterations.** Individual DTI metrics are presented according to lesion subtype and treatment group (plain line: treated; dashed line: non-treated) at day 4 (D4) and week 5 (W5) post-surgery. **a.** FA ipsilateral side: corticostriatal and striatal lesions; **b.** FA contralateral side: corticostriatal and striatal lesions; **c.** MD ipsilateral side: corticostriatal and striatal lesions; **d.** MD contralateral side: corticostriatal and striatal lesions; **e.** AD ipsilateral side: corticostriatal and striatal lesions; **f.** AD contralateral side: corticostriatal and striatal lesions; **g.** RD ipsilateral side: corticostriatal and striatal lesions; **h.** RD contralateral side: corticostriatal and striatal lesions; **i.** FA ipsilateral side: hypothalamic lesions; **j.** FA contralateral side: hypothalamic lesion; **k.** MD ipsilateral side: hypothalamic lesions; **l.** MD contralateral side: hypothalamic lesion; **m.** AD ipsilateral side: hypothalamic lesions; **n.** AD contralateral side: hypothalamic lesion; **o.** RD ipsilateral side: hypothalamic lesions; **p.** RD contralateral side: hypothalamic lesion; FA: fractional anisotropy, MD: mean diffusivity, AD: axial diffusivity, RD: radial diffusivity.


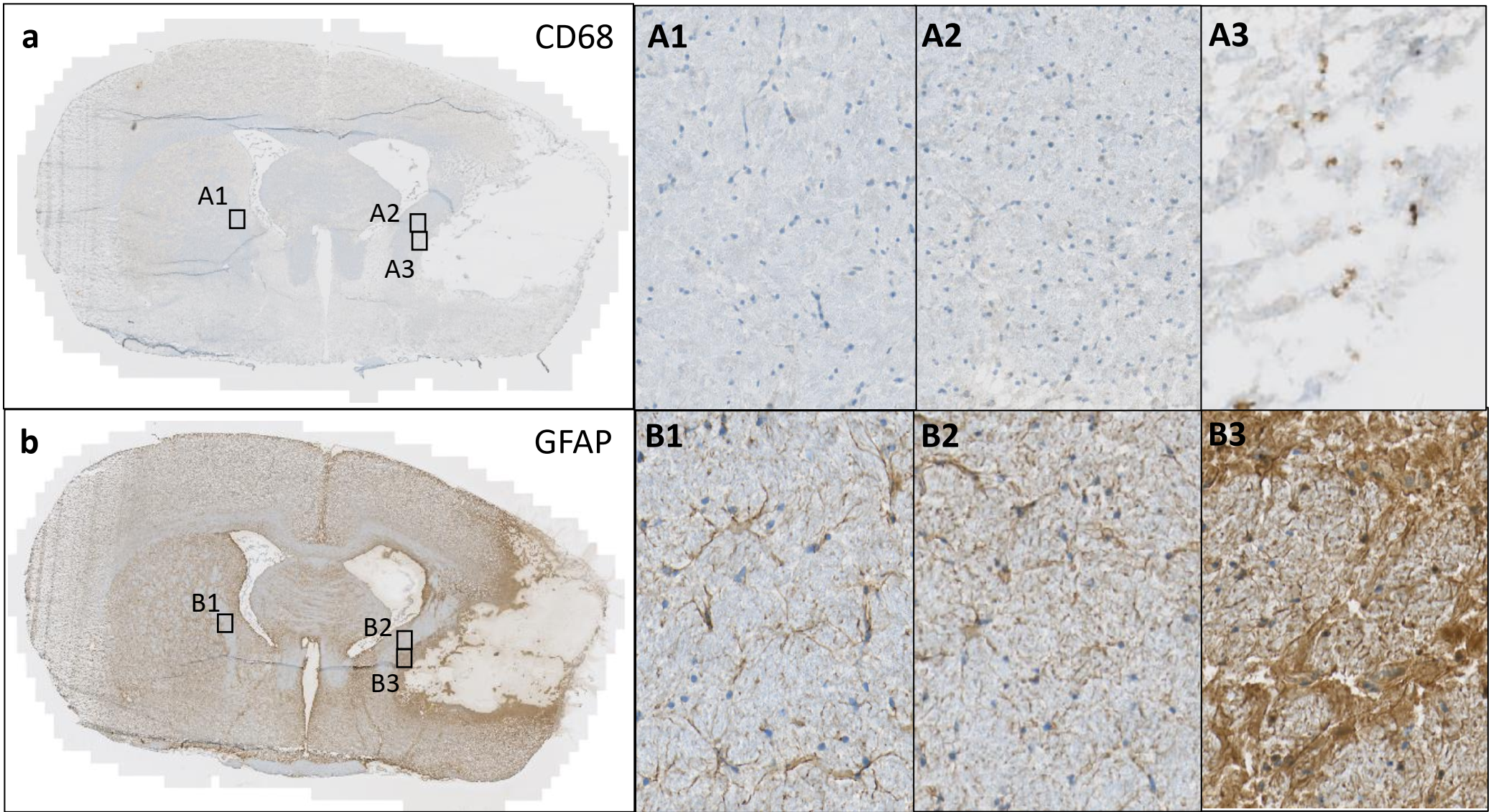


**Supplementary Figure 5- Immunohistochemistry. a.** CD68 immunostaining; A1: contralateral internal capsule; A2: ipsilateral internal capsule; A3: perilesional area; **b.** GFAP immunostaining; B1: contralateral internal capsule; B2: ipsilateral internal capsule; B3: perilesional area.


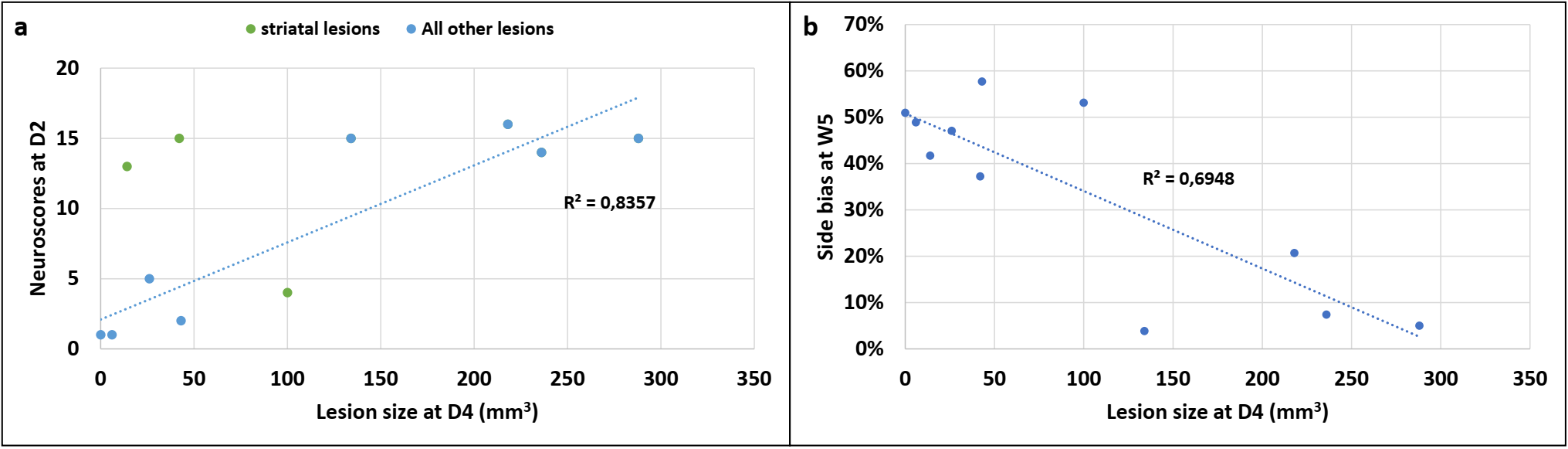


**Supplementary Figure 6- Relationship between lesion size and neurofunctional outcome. a.** Linear relationship between neuroscores at D2 and lesion size at D4; **b.** Linear relationship between side bias at W5 and lesion size at D4.
